# LncRNA FOXD3-AS1/miR-135a-5p function in nasopharyngeal carcinoma cells

**DOI:** 10.1515/med-2020-0177

**Published:** 2020-11-28

**Authors:** Zhang E, Chunli Li, Yuandi Xiang

**Affiliations:** Department of Otorhinolaryngology, Wuhan No. 1 Hospital, No. 215 Zhongshan Road, Wuhan 430022, China

**Keywords:** LncRNA FOXD3-AS1, nasopharyngeal carcinoma cells, miR-135a-5p

## Abstract

This research aimed to illustrate the biological function and associated regulatory mechanism of lncRNA FOXD3-AS1 (FOXD3-AS1) in nasopharyngeal carcinoma (NPC). This research initially found that FOXD3-AS1 was obviously upregulated in NPC cell lines by quantitative reverse transcription polymerase chain reaction (qRT-PCR) detection. Next, the direct target of FOXD3-AS1 was predicted by bioinformatics and further verified by dual-luciferase reporter assay. MiroRNA-135a-5p (miR-135a-5p) was identified as the target gene of FOXD3-AS1 and down-expressed in C666-1 cells compared to NP69. In addition, function assays were conducted in C666-1 cells, including methyl tetrazolium assay, flow cytometry, Caspase3 activity detection, and western blot assay. Our results suggested that miR-135a-5p upregulation inhibited NPC cell growth, enhanced cell apoptosis, promoted Caspase3 activity, increased cleaved-Caspase3, and reduced pro-Caspase3 level. Moreover, we found that FOXD3-AS1 knockdown notably inhibited C666-1 cell proliferation, increased cell apoptosis, enhanced Caspase3 activity, enhanced cleaved-Caspase3 expression, and suppressed pro-Caspase3 level in C666-1 cells. However, these findings were reversed in C666-1 cells by miR-135a-5p mimic co-transfection. To sum up, our data showed that FOXD3-AS1 knockdown regulated cell growth and apoptosis in NCP cells via altering miR-135a-5p expression, suggesting that FOXD3-AS1 might be a therapeutic target for NPC diagnosis and treatment.

## 1 Introduction

Nasopharyngeal carcinoma (NPC), an epithelial malignancy, is related to Epstein–Barr virus infection [1]. The pathogenesis of NPC is multifactorial, such as genetic susceptibility [2], social practices, and environmental factors [3,4]. NPC is frequent in southern China and Southeast Asia with an incidence of 40 cases per 1,00,000 individuals yearly; however, it is infrequent in Western Europe and North America [5]. Although many improvements have been made in NPC treatment, such as radiotherapy and chemotherapy, patients with advanced metastases in NPC have poor clinical symptoms and survival rate [6]. Therefore, investigating the molecular mechanisms would benefit to the research of new therapies for NPC.

In recent years, relationship between lncRNAs and diseases has been evidenced, which inspired us to explore new biomarkers and therapies [7]. LncRNAs are a class of non-coding RNAs, which have more than 200 nucleotides without protein encoding ability [8]. Moreover, various reports have confirmed that lncRNAs played vital regulatory roles in biological processes, including cell proliferation, apoptosis, migration, and invasion [9,10]. LncRNA FOXD3-AS1 (FOXD3-AS1), a novel target researched, was expressed abnormally in many cancers. For instance, Chen et al. reported that FOXD3-AS1 enhanced cutaneous malignant melanoma cell proliferation, invasion, and migration by regulating miR-325/MAP3K2 [11]. Besides, Guan et al. found that FOXD3-AS1 is related to clinical development and regulates breast cancer cell migration and invasion [12]. However, the potential roles of FOXD3-AS1 in NPC have not been lucubrated. Therefore, our study evaluated FOXD3-AS1 expression and role in NPC cells via knocking down FOXD3-AS1 expression.

MiRNAs are endogenous and non-coding RNA molecules, which can mediate target gene levels by directly targeting the 3′-UTR of target mRNAs [13]. Studies have revealed that lncRNAs were dysregulated in multiple biological processes by sharing miRNAs [14]. Moreover, aberrant expression of miRNAs is closely related to the progression of malignant tumors, indicating the potential functions for disease diagnosis and treatment. For example, Dong et al. found that dysregulation of miRNAs altered biogenesis procedure in bladder cancer [15]. Previously, various miRNAs have been confirmed to be abnormally expressed in NPC and involved in the progression of NPC, including miR-100 [16], miR-214 [17], and miR-373 [18]. Previous study has illustrated that miroRNA-135a-5p (miR-135a-5p) was the main regulator in tumor and upregulated or downregulated in cells or tissues [19]. Gao et al. found that miR-135a-5p affects the differentiation of human adipose-derived mesenchymal stem cells by regulating Hippo signaling pathway [20]. Yet, the role of miR-135a-5p in NPC remains unclear.

Therefore, the current research explored the levels of FOXD3-AS1 and its biological functions in NPC cells. The influences of FOXD3-AS1 downregulation on cell proliferation and apoptosis were further illustrated in transfected NPC cells. Furthermore, it was explored whether miR-135a-5p was a direct target of FOXD3-AS1 in NPC. Results indicated that FOXD3-AS1 knockdown exerts antitumor effect in NPC via regulating miR-135a-5p, which will provide further understanding of the molecular mechanisms and therapeutic strategies for NPC treatment.

## 2 Materials and methods

### 2.1 Cell culture

The normal nasopharyngeal epithelial cell line NP69 and NPC cell line C666-1 were achieved from American Type Culture Collection (ATCC, USA). The cells were cultivated in medium RPMI 1640 (Invitrogen; USA) containing 15% fetal bovine serum (FBS; Gibco, USA) and 1% penicillin/streptomycin (Gibco; USA) in an incubator containing 5% CO_2_ at 37°C.

### 2.2 Cell transfection

The mimic control, miR-135a-5p mimic, FOXD3-AS1-siRNA, control-siRNA, and miR-135a-5p inhibitor or inhibitor control were transfected into C666-1 cells by Lipofectamine 2000 (Life Technologies Corporation, USA) for 48 h referring to the manufacturer’s protocol.

### 2.3 Dual-luciferase reporter assay

The binding sites of FOXD3-AS1 and miR-135a-5p were predicted using Starbase version 3.0 (http://starbase.sysu.edu.cn/), which was confirmed using dual luciferase reporter analysis. The FOXD3-AS1 binding sites on miR-135a-5p, containing wild-type or mutant, were inserted into the pmirGLO vector to establish luciferase reporter vector. Then mimic control and miR-135a-5p mimic (GenePharma, China) were co-transfected with luciferase reporter plasmids (FOXD3-AS1-WT and FOXD3-AS1-MUT) into 293T cells using lipofectamine 2000 (Invitrogen) following the manufacturer’s protocols. At 48 h after transfection, the luciferase activity was assessed using dual luciferase reporter kit (KeyGen).

### 2.4 Detection of Caspase-3 activity

The Caspase-3 activity in transfected C666-1 cells was evaluated using Caspase-3 Assay Kit (Beyotime, China) referring to the manufacturer’s instruction. In brief, transfected cells were dissolved with buffer solution, and then lysate was collected and centrifuged for 10 min at 1,500 × *g*. Then 100 mL of Caspase-3 reagent was added to the supernatant and incubated at 37°C for 2 h. After that, optical density (OD) at 405 nm was measured using microplate reader (BioTek, VT) to determine Caspase-3 activity.

### 2.5 Methyl tetrazolium assay

For methyl tetrazolium (MTT) assay, C666-1 cells were incubated in 96-well plates and transfected with mimic control, miR-135a-5p mimic, FOXD3-AS1-siRNA, control-siRNA, and miR-135a-5p inhibitor or inhibitor control for 48 h and cultured for 0, 12, 24, 48, and 96 h at 37°C. After incubation, 20 μL MTT solution was added to the cells, followed by another 4 h incubation. Then 150 μL DMSO was applied to dissolve lysate for 10 min, followed by OD detection at 490 nm using multifunctional plate reader (BIOTEK, USA).

### 2.6 Flow cytometry analysis

Apoptosis of C666-1 cells were measured using flow cytometry assay. C666-1 cells were transfected with mimic control, miR-135a-5p mimic, FOXD3-AS1-siRNA, control-siRNA, and miR-135a-5p inhibitor or inhibitor control for 48 h. Then transfected cells were collected and stained using Annexin V-FITC and PI for 15 min referring to the manufacturer’s protocol. Finally, the apoptotic cells were quantified by flow cytometer (BD Biosciences, USA). Data were analyzed using FlowJo software (version 7.6.1; FlowJo LLC).

### 2.7 qRT-PCR

Total RNA of NP69 and NPC cell line C666-1 was extracted using TRIzol reagent (Invitrogen) referring to the manufacturer’s guidance. cDNA Reverse Transcription Kit (Fermentas, USA) was applied to reversely transcribe RNA to cDNA. The FOXD3-AS1 and miR-135a-5p levels were assessed using ABI SYBR®Green PCR Master Mix (Takara, China). GAPDH and U6 were used as internal reference for mRNA or miRNA, respectively.

The primer used is as follows:

GAPDH-forward, 5′-GGGGCTCTCCAGAACATC-3′;

reverse, 5′-TGACACGTTGGCAGTGG-3′;

U6-forward, 5′-AATTTGAAGAAGCGGTTGC-3′;

reverse, 5′-GTGGAACTGGGAGAACAAG-3′;

FOXD3-AS1-forward, 5′-CCTTCGGCTCACAGCTC-3′;

reverse, 5′-GCTTGGCTCGGACTTGAT-3′;

miR-135a-5p-forward, 5′-TTGAAGAAACCCTTGAGGAA-3′;

reverse, 5′-CTGCCGAATAATCTCCATCT-3′.

Target gene expression was calculated using 2^−ΔΔCq^ method.

### 2.8 Western blot analysis

Total proteins from transfected C666-1 cells were extracted and valued using BCA Protein Assay Kit (Invitrogen, USA). Then averaged proteins were split in 10% SDS–PAGE and transferred to PVDF membrane (Millipore, USA). After that, the membranes were blocked with 5% nonfat milk at room temperature for 2 h and hatched in primary antibodies against GAPDH, cleaved-Caspase 3, and pro-Caspase3 (CST; at a 1:2,000 dilution) overnight at 4°C. Then the membranes were placed in corresponding secondary antibodies (Abcam; 1:2,000 dilution) at room temperature for 1 h. Finally, the target bands were assessed by ECL detection system reagents (Millipore, MA, USA) based on the manufacturer’s instructions.

### 2.9 Statistical analysis

Statistical analysis was performed using SPSS 21.0 (SPSS, USA). Statistical values were presented as mean ± standard deviation from three repeated experiments. Measurement differences among groups were analyzed by the one-way analysis of variance (ANOVA) with Tukey’s *post hoc* test or Student’s *t*-test. *P*-values <0.05 are statistically significant.

## 3 Results

### 3.1 FOXD3-AS1 and miR-135a-5p expression in NPC cells

**Figure 1:**
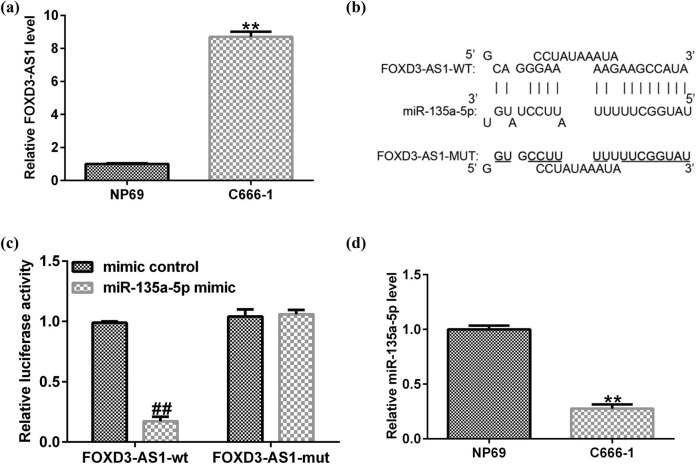
miR-135a-5p and FOXD3-AS1 expression in NPC cells. (a) mRNA expression of FOXD3-AS1 in NP69 and C666-1 cells. (b) Prediction of binding site between miR-135a-5p and FOXD3-AS1 by bioinformatics software. (c) The target relationship between miR-135a-5p and FOXD3-AS1 was confirmed by dual luciferase reporter assay. (d) Relative expression of miR-135a-5p in NP69 and C666-1 cells was assessed using qRT-PCR. ^**^ *P* < 0.01 compared to NP69; ^##^ *P* < 0.01 compared to mimic control.

The level of FOXD3-AS1 in normal nasopharyngeal epithelial cell line NP69 and NPC cell line C666-1 was measured using quantitative real-time polymerase chain reaction (qRT-PCR). As shown in Figure 1a, FOXD3-AS1 was overexpressed in NPC cell line C666-1. To explore the potential mechanisms, bioinformatics analysis was conducted to reveal the predicted miRNAs, which may target FOXD3-AS1. Bioinformatics analysis predicted the targeting sequence between FOXD3-AS1 and miR-135a-5p (Figure 1b).

Previous research has demonstrated that miR-135a-5p directly targeted FOXD3-AS1. Then dual-luciferase reporter analysis was applied to verify this finding. The results in Figure 1c revealed that the luciferase activity of FOXD3-AS1-wt was substantially reduced, whereas the FOXD3-AS1-mut group exerted no impact as compared to control. These results indicated that FOXD3-AS1 directly targeted to miR-135a-5p. Next, we detected miR-135a-5p levels in NP69 and NPC cell line C666-1 cells. Our results from qRT-PCR indicated that miR-135a-5p was expressed significantly low in NPC cell line C666-1 compared to NP69 cells (Figure 1d). These findings clarified that FOXD3-AS1 had direct binding sites with miR-135a-5p and was involved in the development of NPC.

### 3.2 The effects of miR-135a-5p mimic on C666-1 cell proliferation and apoptosis

**Figure 2:**
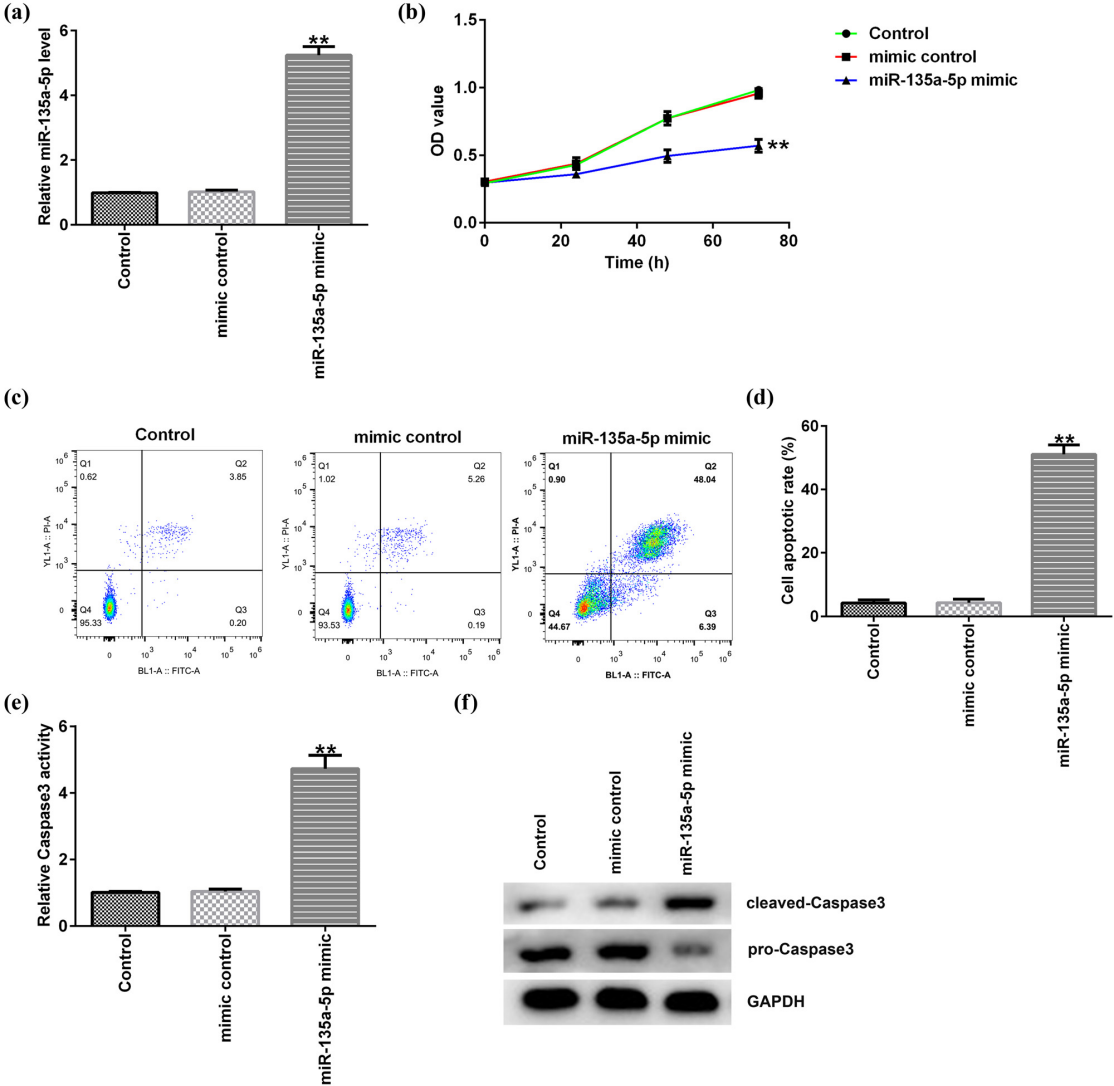
Effects of miR-135a-5p on NPC cell proliferation and apoptosis. (a) The expression of miR-135a-5p in C666-1 cells was determined by qRT-PCR. (b) C666-1 cell proliferation was measured using MTT assay. (c) C666-1 cell apoptosis in miR-135a-5p mimic group and mimic control group was quantified by flow cytometry. (d) The percentage of apoptotic C666-1 cells was calculated and presented. (e) Caspase-3 activity in C666-1 cells was determined. (f) Protein expression of cleaved-Caspase3 and pro-Caspase3 was detected by western blot assay. ^**^ *P* < 0.01 compared to mimic control.

To explore the role of miR-135a-5p in C666-1 cells, mimic control or miR-135a-5p mimic were transfected into C666-1 cells for 48 h. The results in Figure 2a showed that miR-135a-5p was upregulated in C666-1 cells compared to mimic control. In addition, MTT and flow cytometry assay were conducted to determine the C666-1 cell proliferation and quantified the apoptotic cells. We found that C666-1 cell proliferation was inhibited by miR-135a-5p mimic (Figure 2b). Besides, overexpression of miR-135a-5p induced more apoptotic cells (Figure 2c, d). These data demonstrated that miR-135a-5p might be involved in NPC progression. Then we illustrated the potential mechanism by which miR-135a-5p mediated NPC cell apoptosis. Compared to mimic control group, miR-135a-5p mimic obviously advanced the Caspase-3 activity in miR-135a-5p mimic-treated C666-1 cells (Figure 2e). Moreover, the apoptotic-related protein cleaved-Caspase3 was enhanced, and pro-Caspase3 level was inhibited in C666-1 cells by miR-135a-5p mimic (Figure 2f). These results indicated that miR-135a-5p might be participated in NPC development through regulating NPC cell proliferation and apoptosis.

### 3.3 MiR-135a-5p inhibitor abolished the effects of FOXD3-AS1-siRNA on miR-135a-5p expression in C666-1 cells

**Figure 3:**
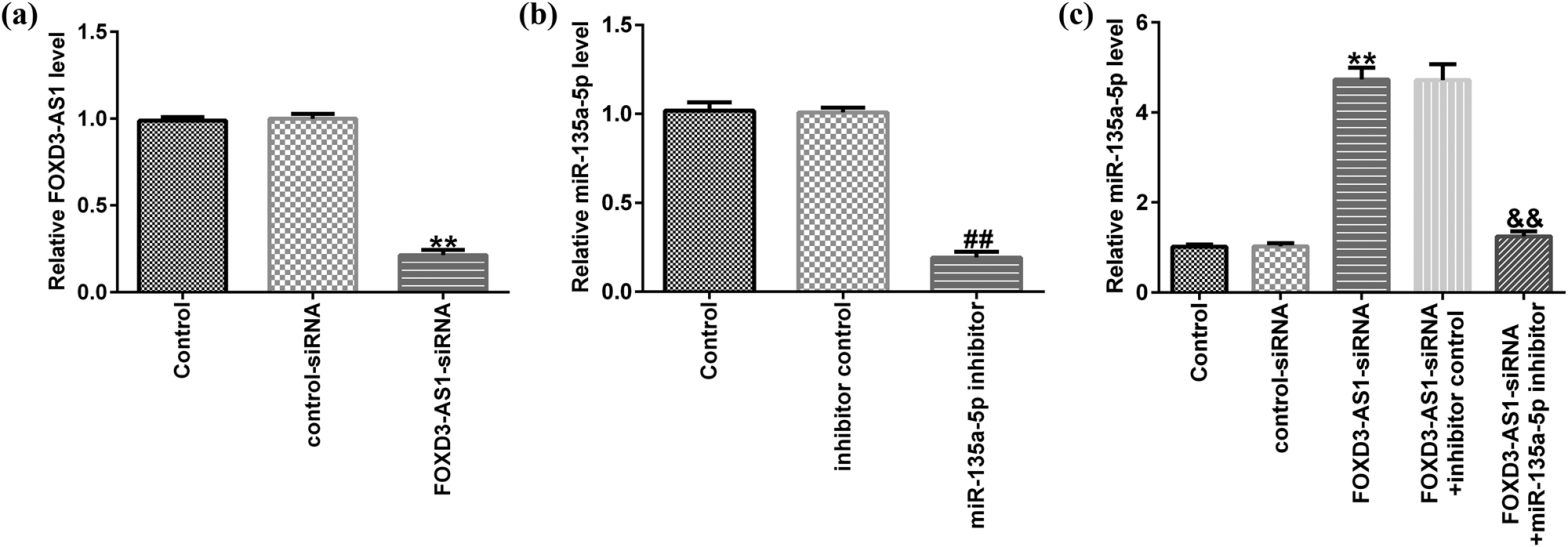
Effects of FOXD3-AS1-siRNA on miR-135a-5p expression in C666-1 cells. (a) The levels of FOXD3-AS1 were determined using qRT-PCR in control-siRNA or FOXD3-AS1-siRNA transfected cells. (b) qRT-PCR analysis of miR-135a-5p in inhibitor control or miR-135a-5p inhibitor–treated C666-1 cells. (c) qRT-PCR assay was conducted to assess miR-135a-5p expression in FOXD3-AS1-siRNA and miR-135a-5p inhibitor co-transfected C666-1 cells.^**^ *P* < 0.01 vs control-siRNA; ^##^ *P* < 0.01 vs inhibitor control; ^&&^ *P* < 0.01 vs FOXD3-AS1-siRNA + inhibitor control.

To better explore the regulatory correlation between miR-135a-5p and FOXD3-AS1 in C666-1 cells, rescue assay was conducted. C666-1 cells were transfected with FOXD3-AS1-siRNA, control-siRNA, and miR-135a-5p inhibitor or inhibitor control for 48 h, and qRT-PCR was applied to detect transfection efficiency. As shown in Figure 3a, FOXD3-AS1 was downregulated in FOXD3-AS1-siRNA-transfected C666-1 cells. Besides, compared to inhibitor control group, miR-135a-5p inhibitor suppressed miR-135a-5p expression in C666-1 cells (Figure 3b). Compared with the control-siRNA group, FOXD3-AS1-siRNA significantly enhanced miR-135a-5p expression in C666-1 cells. On the contrary, this improvement was abolished in FOXD3-AS1-siRNA + miR-135a-5p inhibitor co-transfected cells (Figure 3c). Taken together, our findings revealed that FOXD3-AS1 negatively regulated miR-135a-5p expression in NPC cells.

### 3.4 miR-135a-5p inhibitor reversed the effects of FOXD3-AS1-siRNA on cell proliferation and apoptosis in NPC cells

**Figure 4:**
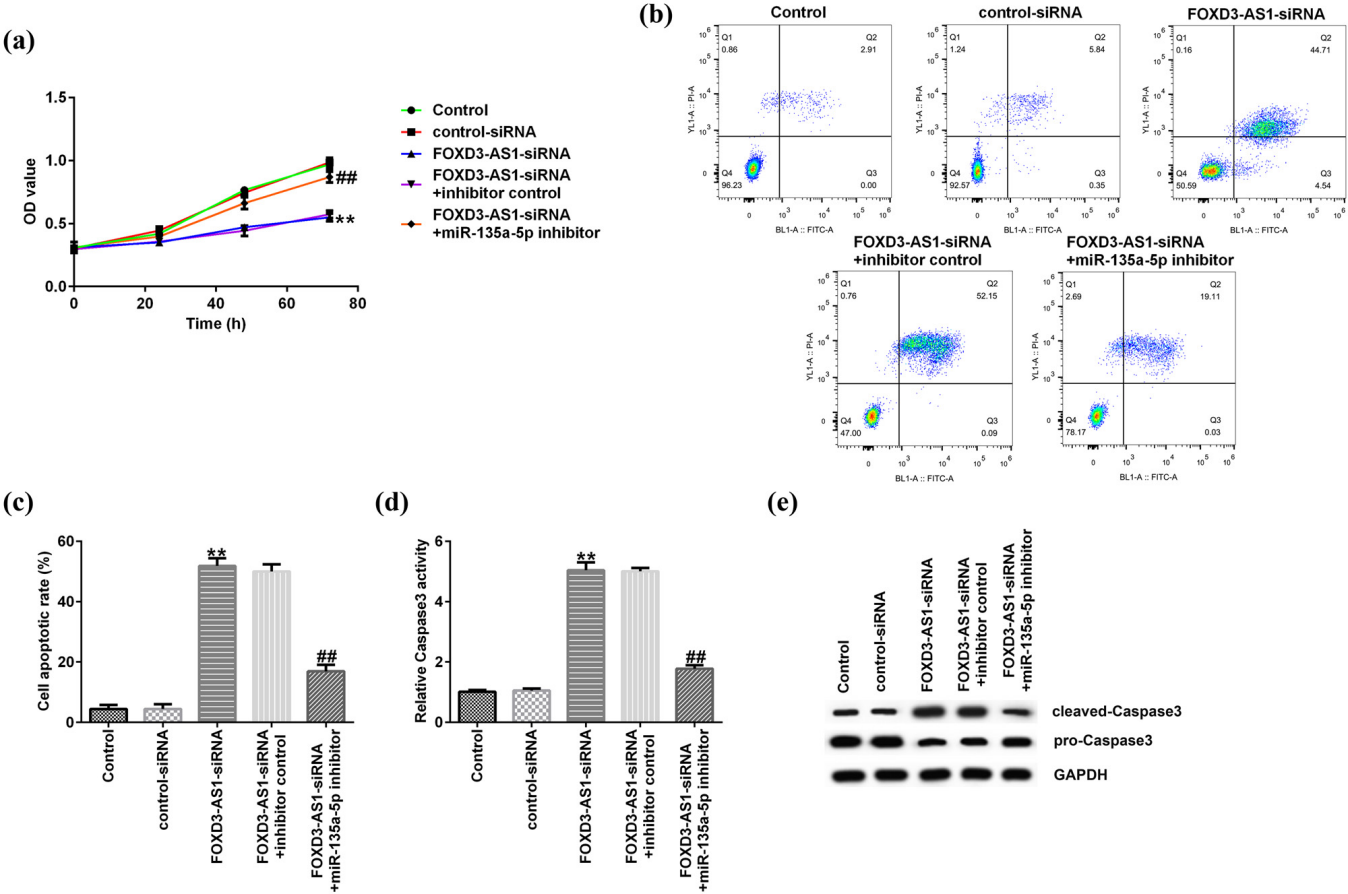
miR-135a-5p inhibitor rescued the influence of FOXD3-AS1-siRNA on C666-1 cells. (a) The proliferation of C666-1 cells was detected using MTT. (b) Detection of apoptosis of C666-1 cells using flow cytometry. (c) Quantification of apoptosis of C666-1 cells. (d) Caspase-3 assay was applied to evaluate Caspase-3 activity in C666-1 cells. (e) The protein expression of cleaved-Caspase3 and pro-Caspase3 was analyzed through western blot assay. ^**^ *P* < 0.01 vs control-siRNA; ^##^ *P* < 0.01 vs FOXD3-AS1-siRNA + inhibitor control.

The function of FOXD3-AS1 in NPC cells was further explored, and MTT and flow cytometry assay were applied to assess NPC cell proliferation and apoptosis, respectively. FOXD3-AS1-siRNA, control-siRNA, and miR-135a-5p inhibitor or inhibitor control were transfected into C666-1 cells. As shown in Figure 4a, FOXD3-AS1-siRNA markedly decreased cell proliferation compared to the control-siRNA group, and this reduction was reversed by miR-135a-5p inhibitor (Figure 4a). Furthermore, flow cytometry revealed that FOXD3-AS1-siRNA significantly promoted the apoptosis rates of C666-1 cells, whereas miR-135a-5p inhibitor rescued the effects of FOXD3-AS1-siRNA on C666-1 cells (Figure 4b, c). In addition, our data in Figure 4d revealed that FOXD3-AS1-siRNA significantly enhanced the activity of Caspase3, and this increase was inhibited by miR-135a-5p inhibitor. Meanwhile, we observed that the expression of cleaved-Caspase3 was increased and the level of pro-Caspase3 was decreased in C666-1 cells by the knockdown of FOXD3-AS1. However, these results were partially reversed by miR-135a-5p inhibitor (Figure 4e). Thus, our findings indicated that knockdown of FOXD3-AS1 depressed cell proliferation and promoted apoptosis in NPC cells via upregulating miR-135a-5p expression, which could block the progression of NPC.

## 4 Discussion

NPC is an uncommon malignancy in most areas of the world. NPC has been a scabrous problem in southern China because of recurrence and distant metastasis [21]. In addition, previous report has demonstrated that the clinical prognosis of NPC was related to Epstein–Barr virus infection [22,23]. Several reports have demonstrated that many genes were regulated in NPC via Epstein–Barr virus infection. For example, Huo et al. found that CRISPR/Cas9-mediated LMP1 knockout could inhibit Epstein–Barr virus infection and suppress NPC cell proliferation [24]. Deng et al. reported that aberrant SATB1 expression is associated with Epstein–Barr virus infection, metastasis, and survival in human nasopharyngeal cells and endemic NPC [25]. However, the detailed pathogenesis of NPC has not been fully understood.

Accumulating studies have found that lncRNA exerted vital regulation roles in the occurrence and progression of diseases [26]. In addition, many lncRNAs have excellent antitumor effects on tumor growth *in vitro* and *in vivo* [27]. FOXD3-AS1 is a novel lncRNA that has been found to mediate cell proliferation, invasion, and apoptosis in many cancers, including NPC. Xu et al. reported that FOXD3-AS1 was up-regulated in NPC [28]. Report from Chen et al. showed that FOXD3-AS1 knockdown suppressed cell proliferation, migration, and invasion in malignant glioma cells [29]. However, the precise mechanism and complex pathogenesis of FOXD3-AS1 in NPC need to be further explored, and there were no effective therapies for NPC patients. Therefore, further investigation of differently expressed lncRNA in NPC will help to illustrate the underlying mechanisms of NPC and find new methods for NPC diagnosis and treatment.

First, FOXD3-AS1 expression in NPC was determined by qRT-PCR. Our results suggested that FOXD3-AS1 expression in C666-1 cells was remarkably higher than that in nasopharyngeal epithelial cell line NP69. Results from other researches proved that FOXD3-AS1 participated in cell biological behaviors via regulating miRNAs, such as miR-296-5p [30], miR-325 [31], and miR-135a-5p [32]. In addition, many miRNAs were confirmed to be involved in the progression of diseases and serve as diagnostic markers. MiR-135a-5p, a conservative gene, has been reported to be expressed abnormally in human cancers, including thyroid carcinoma [33] and head neck squamous cell carcinoma [34]. Besides, miR-135a-5p could participate in cell biological course and alter other protein levels, which may be a meritorious biomarker in the diagnosis of diseases. Liu et al. showed that miR-135a-5p dysregulation promoted the development of rat pulmonary arterial hypertension *in vivo* and *in vitro* [35]. Previous study has confirmed that miR-135a-5p directly targeted FOXD3-AS1 [32]. In this study, bioinformatics assay was used to predict the potential target genes of FOXD3-AS1, and the relationship between FOXD3-AS1 and miR-135a-5p was confirmed using a dual luciferase reporter system. Our results were similar to the previous research and revealed that miR-135a-5p directly targeted FOXD3-AS1. Furthermore, results from qRT-PCR indicated that miR-135a-5p was downregulated in C666-1 cells compared to NP69. Consequently, these findings revealed that FOXD3-AS1 was involved in NPC by negatively regulating miR-135a-5p.

miRNAs dysregulation is a frequent phenomenon in many diseases, which drives cancer development [35]. To illustrate whether miR-135a-5p impacts the development of NPC, mimic control or miR-135a-5p mimic were transfected into C666-1 cells, and we further valuated the effects of miR-135a-5p mimic on biological behavior in NPC cells. Our findings demonstrated that miR-135a-5p mimic effectively suppressed cell proliferation and increased apoptotic cells, making miR-135a-5p as a new therapeutic target in NPC investigation. In addition, we then explored the potential mechanism by which miR-135a-5p mediated NPC cells apoptosis. We found that miR-135a-5p mimic obviously enhanced the Caspase-3 activity in C666-1 cells. Moreover, the apoptotic-related protein cleaved-Caspase3 was enhanced, and pro-Caspase3 level was attenuated in C666-1 cells by miR-135a-5p mimic. Altogether, these results indicated that miR-135a-5p might exert inhibition effect on C666-1 cell proliferation.

To better illustrate the regulatory correlation between miR-135a-5p and FOXD3-AS1 in NPC, C666-1 cells were transfected with FOXD3-AS1-siRNA, control-siRNA, and miR-135a-5p inhibitor or inhibitor control for 48 h. As presented in qRT-PCR analysis, FOXD3-AS1 was downregulated in FOXD3-AS1-siRNA-transfected C666-1 cells, and miR-135a-5p inhibitor suppressed miR-135a-5p expression in C666-1 cells. Moreover, FOXD3-AS1-siRNA enhanced miR-135a-5p expression, and this promotion was eliminated in FOXD3-AS1-siRNA + miR-135a-5p inhibitor co-transfected cells. As we all know, dysregulation of miRNA may affect the biological behaviors of cancer cells [36]. Similar findings were reported by Zhang et al., who demonstrated that dysregulation of miR-202-3p affects migration and invasion of endometrial stromal cells in endometriosis via targeting ROCK1 [37]. Therefore, we explored the roles of miR-135a-5p inhibitor or FOXD3-AS1-siRNA in C666-1 cell biological behaviors. In this study, knockdown of FOXD3-AS1 attenuated C666-1 cell proliferation and improved cell apoptosis. Many targets exert functions in tumorigenesis and development of diseases by influencing signal pathways and regulating genes, such as Caspase-3, which directly influenced cell apoptosis [38]. Similar results were observed in this research: FOXD3-AS1-siRNA promoted Caspase-3 activity, enhanced cleaved-Caspase3 levels, and suppressed pro-Caspase3 expression. However, inhibition of miR-135a-5p could rescue the effects of FOXD3-AS1-siRNA on C666-1 cells, but there were some limitations to this study. For example, only one cell line (C666-1 cells) was used in this study and more cell lines should be investigated. The target of miR-135a-5p was not determined in this study. Moreover, we only investigated the role of FOXD3-AS1 in NPC *in vitro*, and *in vivo* experiments needed to be performed to reveal the role of FOXD3-AS1 in NPC. We will further investigate these issues in the future.

Taken together, these results revealed that FOXD3-AS1 knockdown could suppress cell proliferation and induce cell apoptosis by upregulating miR-135a-5p expression in NPC cells. Our findings broadened our understanding of the relevance between lncRNAs and miRNAs. In addition, we provided new targets for the diagnosis and treatment of NPC.
